# Measuring transverse relaxation in highly paramagnetic systems

**DOI:** 10.1007/s10858-020-00334-w

**Published:** 2020-07-24

**Authors:** Michele Invernici, Inês B. Trindade, Francesca Cantini, Ricardo O. Louro, Mario Piccioli

**Affiliations:** 1 Instituto de Tecnologia Química e Biológica António Xavier (ITQB-NOVA), Universidade Nova de Lisboa, Av. da República (EAN), 2780-157 Oeiras, Portugal; 2 Magnetic Resonance Center (CERM) and Department of Chemistry, University of Florence, Via L. Sacconi 6, 50019 Sesto Fiorentino, Italy; 3 Consorzio Interuniversitario Risonanze Magnetiche Di Metallo Proteine (CIRMMP), Via L. Sacconi 6, 50019 Sesto Fiorentino, Italy

**Keywords:** Paramagnetic NMR, Iron sulfur proteins, Pulse sequences, NMR based structural restraints, Transverse relaxation, Paramagnetic relaxation enhancement

## Abstract

The enhancement of nuclear relaxation rates due to the interaction with a paramagnetic center (known as Paramagnetic Relaxation Enhancement) is a powerful source of structural and dynamics information, widely used in structural biology. However, many signals affected by the hyperfine interaction relax faster than the evolution periods of common NMR experiments and therefore they are broadened beyond detection. This gives rise to a so-called blind sphere around the paramagnetic center, which is a major limitation in the use of PREs. Reducing the blind sphere is extremely important in paramagnetic metalloproteins. The identification, characterization, and proper structural restraining of the first coordination sphere of the metal ion(s) and its immediate neighboring regions is key to understand their biological function. The novel HSQC scheme we propose here, that we termed *R*_2_-weighted, HSQC-AP, achieves this aim by detecting signals that escaped detection in a conventional HSQC experiment and provides fully reliable *R*_2_ values in the range of ^1^H *R*_2_ rates ca. 50–400 s^−1^. Independently on the type of paramagnetic center and on the size of the molecule, this experiment decreases the radius of the blind sphere and increases the number of detectable PREs. Here, we report the validation of this approach for the case of PioC, a small protein containing a high potential 4Fe-4S cluster in the reduced [Fe_4_S_4_]^2+^ form. The blind sphere was contracted to a minimal extent, enabling the measurement of R_2_ rates for the cluster coordinating residues.

## Introduction

The hyperfine interaction between electron and nuclear spins gives rise to additional contributions to chemical shifts and nuclear relaxation, both of which can be used as a source of structural restraints (Piccioli and Turano 2015; Turner et al. 1998). Nowadays, NMR solution structures of paramagnetic macromolecules are obtained by a combination of conventional restraints (Ab et al. 2006; Mori et al. 2008), such as NOE and residual dipolar couplings, and of paramagnetic-based restraints (Arnesano et al. 2006; Clore 2015; Kudhair, et al. 2280; Parigi et al. 2019; Pintacuda et al. 2007). Depending on which paramagnet is present in the system, different combinations of contact shifts, pseudocontact shifts, paramagnetic relaxation enhancements and cross correlation rates can be used (Koehler and Meiler 2011; Clore and Iwahara 2009; Kateb and Piccioli 2003; Pintacuda 2004). However, since about two decades, many groups have promoted the use of paramagnetism-based NMR restraints to study also diamagnetic proteins: metal binding tags have been used as spin-labels and provide various restraints capable to complement the NMR information available on a native, diamagnetic, derivative (Iwahara et al. 2004; Miao 2019; Matei and Gronenborn 2016; Liu et al. 2014; Nitsche and Otting 2017; Joss and Haussinger 2019). NMR of paramagnetic systems is not anymore a playground reserved to scientists working with inorganic or bio-inorganic systems, but a tool for a larger community of structural biologists with many potential applications (Tang et al. 2006; Softley et al. 2020).

Very recently, we have shown that structural restraints derived from Paramagnetic Relaxation Enhancements (PRE) can be used as the unique source of restraints for the structure calculation of small metalloproteins (Trindade et al. 2020b). A key point for a successful PRE-only approach is the availability of many accurate relaxation rate values measured throughout the entire protein, including the close proximity of the paramagnetic center, where nuclear spins are strongly affected by paramagnetism. When hyperfine shifted signals are well isolated outside the bulk diamagnetic envelope, they can be characterized using 1D experiments and relaxation based filters (Inubushi and Becker 1983), although usually only a few signals, arising from the side chains of metal-bound residues, can be identified via this approach (Hansen and Led 2006; Sato et al. 2003; Brancaccio 2017). Paramagnetic relaxation depends on γ^2^ of the observed nucleus, therefore ^13^C or ^15^N direct detection (Arnesano 2003; Kolczak et al. 1999; Lin et al. 2003) have been successful as an efficient alternative to ^1^H detected experiments, not only for assignment purposes (Bermel 2005; Machonkin et al. 2002; Bertini et al. 2005b) but also for the obtainment of PREs. However, the ^15^ N HSQC experiment still remains the “easiest” molecular fingerprint, therefore methods to exploit the use of ^1^H_N_ *T*_1_ and *T*_2_ rates are welcome.

To date, relaxation-based restraints are indeed obtained via ^1^H *T*_1_ and *T*_2_ measurements from ^15^N HSQC-type experiments (Donaldson 2001; Iwahara et al. 2007). However, many signals affected by the hyperfine interaction relax faster than the evolution periods used in these experiments; therefore, they will be broadened beyond detection or, when they are detected, their intensity decay cannot be properly sampled. Thus, we need to design novel pulse sequences to measure *R*_2_ and *R*_1_ rates of resonances that escape detection in conventional HSQC experiments. For longitudinal ^1^H relaxation rates, we have shown that an inversion recovery filtered ^15^N HSQC experiment acquired in antiphase (^15^N IR-HSQC-AP) (Ciofi-Baffoni et al. 2014) is very effective for the identification of signals severely affected by paramagnetism, and also for obtaining *R*_1_ rates faster than those measurable in established experiments. However, it has been reported that ^1^H *R*_2_ values are less susceptible to internal motions and cross relaxation than ^1^H *R*_1_ values (Iwahara et al. 2004; Iwahara and Clore 2010). Therefore, we are interested to develop an experiment to obtain accurate measurements of ^1^H transverse relaxation also in the close proximity of a paramagnetic center.

The experiment we present here has been customized for the case of the HiPIP (High Potential Iron-sulfur Protein) PioC from *Rhodopseudomonas palustris* TIE-1 (Bird, et al. 2014). PioC has 54 amino acids, contains the typical HiPIP binding motif CXXCX_n_CX_m_C and it is the smallest HiPIP isolated so far. Due to the high reduction potential (E^0^ =  + 450 mV vs SHE) of the [Fe_4_S_4_]^3+^/[Fe_4_S_4_]^2+^ redox pair (both oxidation states are paramagnetic), the protein is stable in the reduced [Fe_4_S_4_]^2+^ state. The paramagnetic ^1^H NMR spectrum of PioC is very similar to the NMR spectra of other HiPIPs in the reduced state (Bertini 1992), thus indicating that the electronic relaxation time (τ_e_) in PioC must be similar to previously studied HiPIPs (Banci et al. 2018). However, PioC is the smallest HiPIP isolated so far and about 60% of the protein is affected by paramagnetic relaxation. Counter-intuitively, the small size of the protein makes it more difficult to study, because paramagnetic effects are active in the majority of the protein, scalar and dipolar couplings are quenched and a number of HN signals are not observable in a conventional HSQC experiment (Cheng and Markley 1995; Machonkin et al. 2005; Lin 2009). Throughout this article we will first review why experimental approaches that are effective to study proteins containing metal bindings tags are not equally efficient for native metalloproteins; then we will discuss how implementation of this novel HSQC scheme allows the measurement of relaxation rate values in a range 50–400 s^−1^. Rates in this range were once very difficult to measure reliably, however they are necessary to reduce the blind sphere enabling the characterization, and proper structural restraining of the first coordination sphere of the metal ion(s) and its immediate surrounding residues.

## Materials and methods

### Protein expression and purification

PioC was expressed and purified as previously reported (Bird et al. 2014). Uniformly ^15^N labeled samples of PioC were produced and the expression and purification protocol was identical throughout except in the addition of ammonium sulfate (^15^N_2_, 99%) in the M9 minimal media. BL21 DE3 cells were double transformed with pET32h, a plasmid containing the construct thioredoxin–6xHis–thrombin cleavage site–PioC, and with pDB1281, a plasmid that carries the machinery for the assembly of iron-sulfur clusters. Cells were grown in Luria–Bertani (LB) medium supplemented with 100 mg*dm^−3^ ampicillin and 35 mg*dm^−3^ chloramphenicol until the OD_600nm_ of 0.6 where they were induced with 1.0 mM arabinose and 20 μM FeCl_3_ and 200 μM cysteine were added. Cells were again incubated until the OD_600nm_ of 1 and then harvested and washed in M9 minimal media salts before resuspension in M9 minimal media. Once re-suspended, cells were incubated for one hour before induction with 0.5 mM IPTG. After 4 h cells were harvested by centrifugation and disrupted using a French Press at 1000 psi. The lysate was ultra-centrifuged at 204,709×*g* for 90 min at 4 °C to remove cell membranes and debris and the supernatant was dialyzed overnight against 50 mM potassium phosphate buffer pH 5.8 with 300 mM NaCl before injection in a His-trap affinity column (GE Healthcare). The fraction containing Histag-PioC eluted with 250 mM imidazole and was incubated overnight with Thrombin (GE Healthcare) for digestion. The final purified PioC (His-tag free) was then concentrated from the flow through of a 2nd passage through the His-trap column using an Amicon Ultra Centrifugal Filter (Millipore) with a 3 kDa cutoff. The purity of PioC was confirmed by SDS-PAGE with Blue Safe staining (NzyTech) and by UV–Visible spectroscopy.

### NMR spectroscopy

Measurements of ^1^H *R*_2_ transverse relaxation rates were carried out using 11.7 T Bruker AVANCE 500 equipped with a triple resonance, inverse detection, cryoprobe (TXI). ^1^H_N_ *R*_2_ measurements were obtained from a series of R_2_-weighted ^15^ N-HSQC-AP experiments, developed throughout this article. For each experiment, 256 scans were collected over 256 increments. Acquisition time and recycle delay were 47.1 ms and 150 ms. A series of sixteen R_2_-weighted ^15^N-HSQC-AP experiments was recorded, using INEPT transfer periods of 0.2 ms, 0.4 ms, 0.6 ms, 0.8 ms, 1.2 ms, 1.6 ms, 2.0 ms, 2.4 ms, 2.8 ms, 3.2 ms, 4.0 ms, 4.8 ms, 5.6 ms, 6.4 ms, 8.0 ms, 10.0 ms. Total experimental time was about 58 h. ^1^H_N_ *R*_2_ measurements have been obtained also with the established approach (Donaldson 2001), in which a relaxation delay *T* is inserted into the INEPT building block of an in-phase ^15^ N HSQC experiment. For each experiment, 32 scans were collected over 156 increments, using acquisition time and recycle delay of 47.1 ms and 4 s, respectively. A series of fourteen^15^N-HSQC-IP experiments was recorded using relaxation delays *T* of 13.3 ms, 17.3 ms, 21.3 ms, 33.3 ms, 45.3 ms, 57.3 ms, 69.3 ms, 81.3 ms, 93.3 ms, 117.3 ms, 141.3 ms, 165.3 ms, 205.3 ms and 245.3 ms. A 1400 μs selective ^1^H_N_ inversion pulse was used for ^3^JH_N_H_α_ decoupling. Total experimental time was about 80 h. Both series have been performed using 256 × 1024 data point matrices, over spectral windows of 80.0 ppm × 21.7 ppm. Squared cosine weighting functions and apodization were used in both dimensions prior to FT, spectra dimensions was 512 × 2048 data points. Peak intensities were used to calculate *R*_2_ values, using the equations described in the results section. All relaxation data were analyzed using the Bruker Topspin Dynamics Center.

### NMR assignment of PioC

The backbone NMR assignment of PioC (Trindade et al. 2020a) has been published on Biomolecular NMR Assignment and deposited in the BMRB data bank (ID 34487).

## Results

### State of the art: an overview

**Fig. 1:**
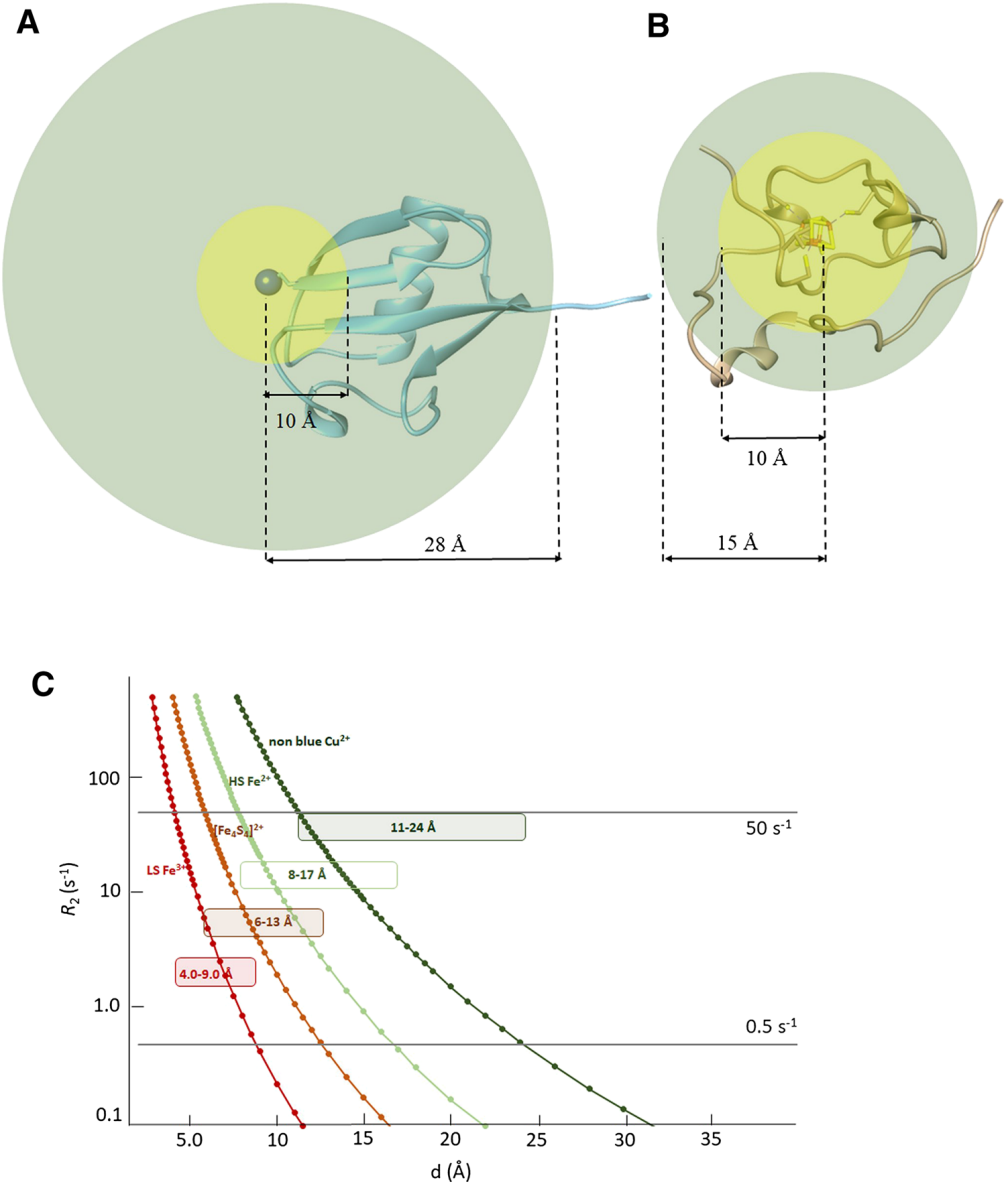
Measurable paramagnetic relaxation enhancements vs metal-to-proton distances. **a** Assuming a 10 Å blind sphere and a 10–30 Å sphere where PREs can be measured, the use of a metal binding tag at the N-term site of Ubiquitin (Donaldson 2001) gives measurable PRE for about 90% of the protein. **b** When the same relaxation enhancement parameters is considered for a small metalloprotein such as HiPIP (Trindade et al. 2020b), more than 70% of the protein would fall in the blind sphere. **c** Different electronic correlation times (τ_e_) provide different dimensions for the regions affected by PREs. Assuming 50 s^1^ and 0.5 s^−1^ as upper and lower limits for the detection of PREs, the simulated behavior of paramagnetic centers with different τ_e_ is shown

The state-of-the-art approach for ^1^H *R*_2_ relaxation is to use a ^15^N HSQC experiment and insert a relaxation period within the INEPT block to measure H_N_ rates (Donaldson 2001). Relaxation rates *R*_2_ can then be obtained either by collecting, for each H_N_ signal, a complete decay curve or by measuring *R*_2_ rates from a two time-point measurement, thereby enabling the direct determination of *R*_2_ values and their associated errors without any fitting procedure (Iwahara et al. 2007). For non-deuterated proteins, band selective ^1^H 180° pulses should be used to avoid the evolution of ^3^*J*H_N_Hα coupling during the relaxation period. The paramagnetic contribution to the overall nuclear relaxation is given (Bertini et al. 2016) by Eq. (1)

1 $$ R_{{2{\text{obs}}}}  = R_{{2{\text{para}}}} +  R_{{2{\text{dia}}}} $$

when *R*_2dia_ and *R*_2obs_ values can be measured with high precision and accuracy (Iwahara et al. 2007), then very small values of paramagnetic relaxation enhancement *R*_2para_ can be obtained by the difference of the two measured values. This has been exploited by attaching a metal binding peptide, such as ATCUN or HHP at the N-terminus site of a diamagnetic protein, as shown in Fig. 1a (Donaldson 2001). Metal binding peptides containing ions with long electronic correlation times, such as, for instance, Cu^2+^ or Mn^2+^, provide paramagnetic relaxation enhancements that can be measured, in the case of Cu^2+^ ion, for all protons located approximately 10–30 Å apart from the metal center (Donaldson 2001; Harford and Sarkar 1997; Jensen et al. 2004; Keizers and Ubbink 2011). The strength of this approach is that, within the above range, both *R*_2obs_ and *R*_2dia_ can be experimentally measured with high accuracy and *R*_2para_ as small as 0.5 s^−1^ can be obtained, thus allowing the use of Paramagnetic Relaxation Enhancement (PRE, hereafter) for long metal-to-proton distance restraints. Protein–protein interaction surfaces and catalytic centers typically fall within this range, therefore one could obtain additional structural information for protein–protein/protein–ligand interactions, structure refinement, etc. (Battiste and Wagner 2000; Hass and Ubbink 2014; Cetiner 2019; Anthis and Clore 2015; Spronk 2018). On the other hand, signals at less than 12 Å from the copper(II) ion will experience *R*_2para_ larger than 50 s^−1^, that will give rise to signals that, in the *R*_2_ experiment, will be very weak and eventually broadened beyond detection. The loss of information typically occurs in a protein region where no catalytic reactions or biochemical events occur, since the metal binding peptide is attached far from the protein core, usually at the N-term site. In metalloproteins, however, the topology of the system is completely different: the metal center(s) and its first coordination sphere always constitute the core region for the protein function. Structural biologists are therefore interested to obtain detailed information in the close proximity of the metal center(s), where the biochemically relevant events occur. Assuming the same paramagnetic relaxation enhancement of the previously described situation, we face a loss of information in the most interesting part of the protein. Such blind region (Balayssac et al. 2006) is, indeed, always the core region of a metalloprotein (Fig. 1B). In this frame, reducing the blind sphere around the paramagnetic center(s) by measuring relaxation rates of nuclear spins that are most affected by paramagnetism becomes extremely important.

The replacement of Cu(II), i.e. the metal ion used in the case of Ubiquitin shown in Fig. 1a, with another metal ion will change the radius of the sphere where paramagnetic relaxation enhancement is effective and measurable, but the above consideration will remain: the metal center is always surrounded by a blind sphere, where signals are broadened beyond detection, and by an outer sphere, in which PREs can be measured and factorized. The situation is described in Fig. 1c, where we considered 50 s^−1^ as upper limit value to obtain reliable *R*_2_ measurements and 0.5 s^−1^ as lower limit for the precision of the measurement. At 500 MHz, considering a protein of small size and neglecting contact relaxation, *R*_2_ is dominated by the Solomon equation (Solomon 1955) which, in turn, depends on the electronic relaxation time. For τ_e_ = 5*10^–9^ s, which is an estimate for the electronic relaxation time of non-blue Cu^2+^ chromophores, ^1^H signals at less than 11 Å are predicted to be unobservable while those at more than 24 Å are almost unaffected by paramagnetism. Different cases may occur for iron ions: for a high spin (S = 2) Fe^2+^, with typical τ_e_ = 5*10^–12^ s, the above limits would be, respectively, 8 Å and 17 Å, while for a low spin (S = 1/2) Fe^3+^, with τ_e_ = 1*10^–12^ s, they would be 4 Å and 9 Å, respectively. Different electronic relaxation times, together with other experimental parameters, such as magnetic field strength and protein size will give rise to different radii for the blind sphere and for the sphere where PREs are measurable. Also the replacement of ^1^H with ^13^C or ^15^ N nuclei reduces both spheres and provides an alternate route to obtain PREs (Mateos et al. 2019). To extend the range of application of PREs in ^1^H detected experiments it is necessary, on the one hand, to increase the accuracy of experimental methods and observe smaller PRE values and, on the other hand, to measure with reliability *R*_2_ values larger than those achievable with the existing experimental approaches.

As extensively studied in the past, [Fe_4_S_4_]^2+^ clusters in proteins have an electronic ground state S = 0 due to the antiferromagnetic coupling (Phillips et al. 1970; Mouesca and Lamotte 1998; Bertini et al. 1992). However, each iron ion is formally in the oxidation state Fe^2.5+^ and the system is paramagnetic at room temperature due to the population of the excited energy levels of the electron spin ladder (Banci et al. 1990). The distribution of population among the spin levels depends on the extent of the antiferromagnetic coupling constant(s) operative in the [Fe_4_S_4_]^2+^ cluster (Blondin and Girerd 1990). Therefore, the choice of the parameters to input in the Solomon equation to predict the behavior of *R*_2_ vs H-Fe distance in [Fe_4_S_4_]^2+^ clusters is not obvious (Bertini et al. 1997): if we consider that each iron ion will relax with the same electronic relaxation time of a High Spin Fe^2+^ ion (τ_e_ = 5*10^–12^), and that paramagnetism at room temperature arises from the population of the first excited state of the electron spin energy ladder, characterized by S = 1, we obtain, as shown in Fig. 1C, a behavior somehow intermediate between the two cases considered here for iron ions, with expected values for the blind sphere and for the PRE sphere of about 6 Å and 13 Å. This makes PioC an ideal test case for the optimization of sequences aiming at measuring *R*_2_ rates in the proximity of the cluster.

### Pulse sequence description

**Fig. 2:**
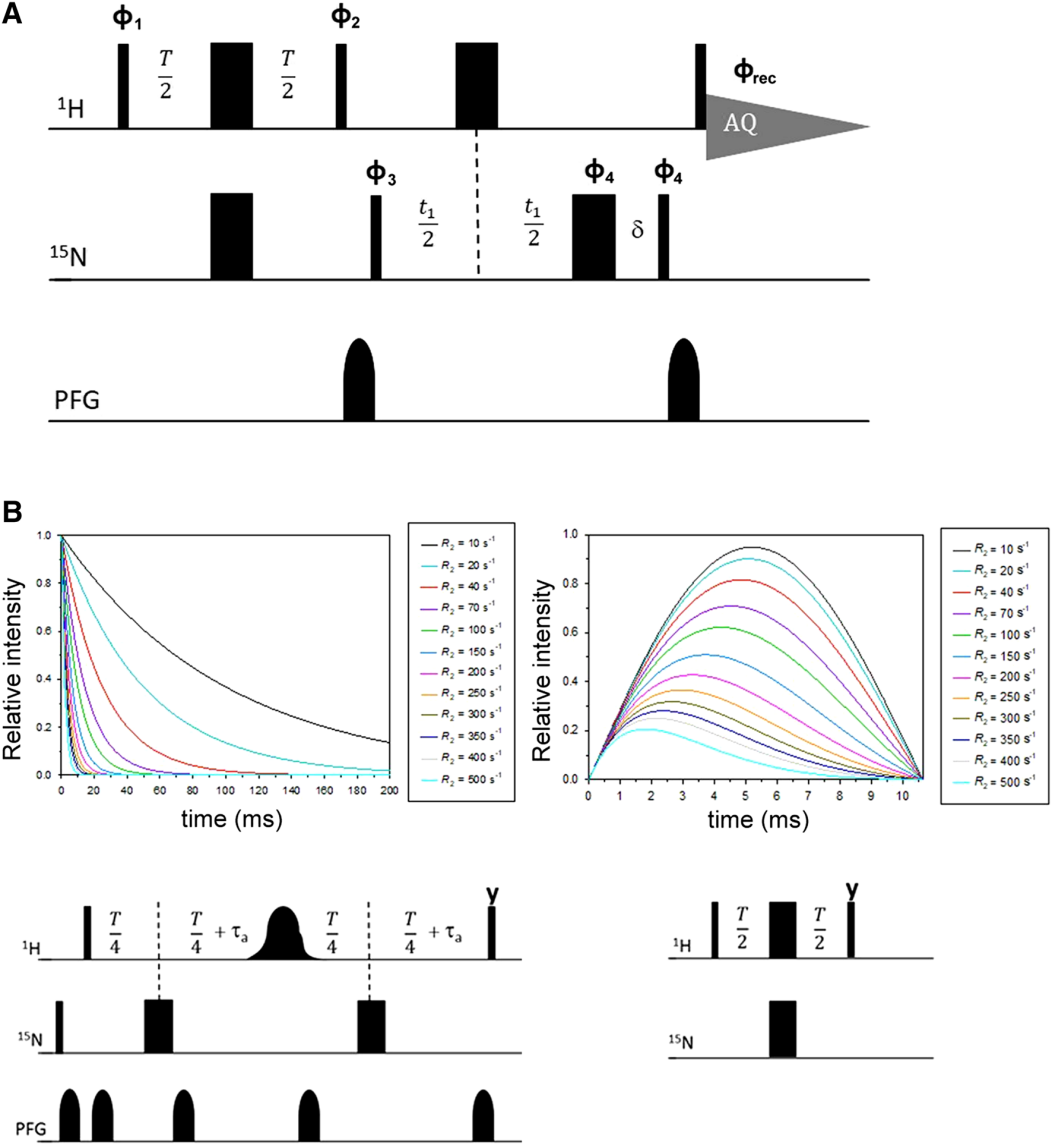
The ^1^H *R*_2_ weighted ^15^N HSQC-AP pulse experiment. **a** Pulse sequence used for the experiment. Hard 90° and 180° pulses are used for both ^1^H and ^15^N channels, using phase x when undefined. Phase cycling: φ_1_ = x, −x, y, −y; φ_2_ = 2(y), 2(x); φ_3_ = 2(x), 2(−x); φ_4_ = 4(x), 4(−x); φ_rec_ = x, −x, −x, x, −x, x, x, −x. PFG gradients of 200 μs were used, with 100 μs for gradient recovery. Standard parameters to measure fast relaxing nuclei as follows: aq = 47 ms, *T* = ranging from 200 μs to 10 ms, recycle delay = 150 ms. **b** Comparison between different *R*_2_ relaxation building blocks. Considering the relaxation building block shown on the left panel (Donaldson 2001; Iwahara et al. 2007), using τ_a_ = 2.65 ms and *T*/4 = 1.8 ms as shortest possible delay, the exponential decay can be measured from about 12.5 ms. Signals with *R*_2_ values over 70 s^−1^ will be lost or detected at very weak intensities for few points. In the *R*_2_-weighted building block shown in the right panel, they could be easily measured for a sufficient number of time delays in the range 0–10 ms, provided signals have enough S/N. The time scales of the two curves highlight the complementarity of the two experiments

Here we propose a novel pulse sequence, shown in Fig. 2a, in which the relaxation delay is embedded within the INEPT evolution, the refocusing INEPT is removed and signal is acquired as antiphase doublet as soon as the H_y_N_z_ magnetization is created by the last ^1^H 90° pulse. We called the experiment ^1^H R_2_-weighted ^15^N-HSQC-AP because the INEPT period, typically 1/(2*J*_HN_), is replaced here by a variable relaxation delay *T*. In this very simple sequence, ^1^H transverse relaxation is active only during the delay *T*, when the 2H_x_N_z_ coherence evolves from H_y_ as H_x_N_z_sin(π*J*_HN_*T*). The relaxation rate of the 2H_x_N_z_ antiphase term is a combination of ^15^ N *R*_1_ and ^1^H *R*_2_ relaxation rates; paramagnetic relaxation rates have a γ^2^ dependence from the observed nucleus (Solomon 1955; Bertini 1986), therefore the contribution of ^15^N *R*_1_ can be neglected and the observed rates are fully due to ^1^H *R*_2_ relaxation (Iwahara et al. 2007). Also cross correlation between ^1^H Curie Spin Relaxation and HN dipole–dipole relaxation is not contributing to the observed rates (Pintacuda et al. 2003; Mori et al. 2010). In order to sample ^1^H relaxation rates also at *T* values of a few μs, we avoid using pulsed field gradients, generally applied during the INEPT period. The delay *T* can then be arrayed from zero to 10 ms to sample the evolution of H_y_ → 2H_x_N_z_ coherence transfer for half a sinusoidal period (1/*J*), during which ^1^H *R*_2_ relaxation is active. To avoid signal losses due to ^1^H *R*_2_ relaxation, the inverse INEPT block is removed and the 2H_y_N_z_ coherence, created by the two 90° pulses at the end of ^15^N evolution, is acquired in antiphase without ^15^N decoupling. Removing the ^15^N decoupling also rules out duty cycle problems: without decoupling, one can safely use very short recycle delays in order to increase S/N of fast relaxing signals and to suppress water signal via progressive saturation (Camponeschi et al. 2019). This may affect the observed relaxation rates of H_N_ signals; however, when the observed values in Eq. (1) are dominated by *R*_2para_, water saturation should not play a significant role. Another very important feature of this sequence is the suitability for cryo-probes, because there are no risks associated to coil heating due to an excess of RF power (Helms and Satterlee 2013). The use of a refocusing INEPT and ^15^N decoupling during acquisition is indeed a severe limiting factor for the recycle delay that, by no means, could have been as short as we used in our experiments.

The *R*_2_-weighted HSQC-AP is essentially the simplest possible scheme for measuring ^1^H *R*_2_ relaxation with an HSQC-type experiment. Figure 2b shows the features of the *R*_2_-weighted building block with respect to the relaxation building block commonly used (Donaldson 2001). For the latter, the relaxation delay *T* must accommodate the selective ^1^H 180° pulse, the Pulsed Field Gradients, the INEPT transfer period 2τ_a_. The shortest possible value of the relaxation delay *T* is set to about 12 ms, indeed this is why very fast relaxing signals are not observed with this approach. The removal of the ^1^H 180° selective pulse and shorter gradients allow one to decrease this value, even though it can’t be below 6.5 ms. As shown in Fig. 2b, for an *R*_2_ rate of 70 s^−1^ signals will be already at 50% of the initial intensity at the first time point of the series, while those exceeding 150 s^−1^ will be beyond detection after two time points of the *R*_2_ series, for all these situations the exponential decay could not be properly analyzed. On the other hand, the *R*_2_-weighted INEPT building block is highly complementary to this approach, because it will monitor the evolution of signal intensities during the first 10 ms of the relaxation recovery, starting from *T* period as small as 100 μs, therefore being able to monitor also very fast relaxing signals. Another interesting feature of the *R*_2_-weighted HSQC is that fast and slow relaxing resonances will be measured with comparable precision.

**Fig. 3:**
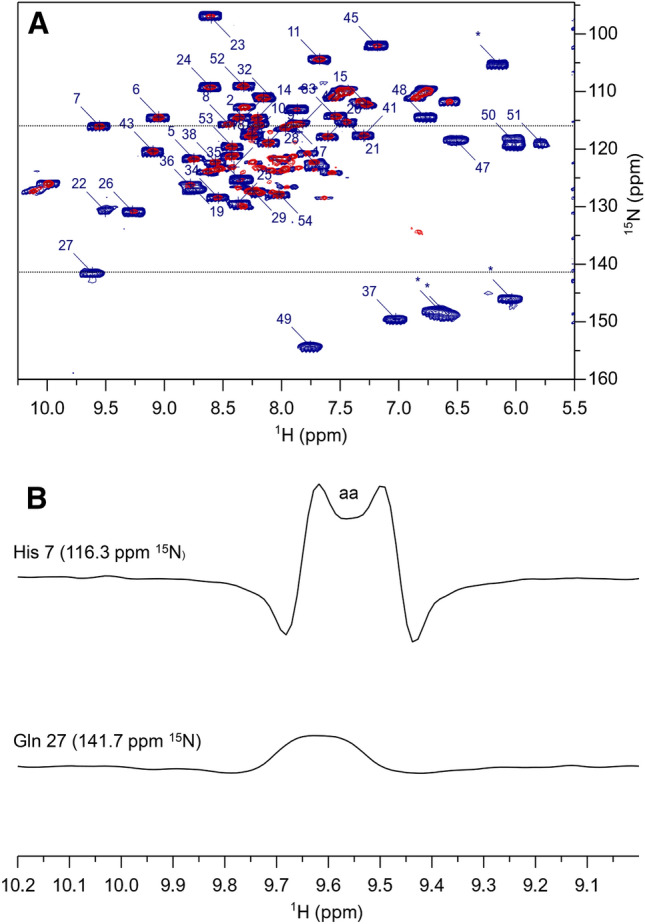
**a** ^15^N HSQC spectra of PioC obtained with an *R*_2_-weighted ^15^N-HSQC-AP experiment collected with T = 2.4 ms (blue) and the first point of the R_2_ series collected with the standard experiment with an overall relaxation delay = 12.5 ms (red). Experiments were recorded using a 500 MHz NEO-Avance Bruker spectrometer equipped with Triple resonances inverse cryoprobe (CP-TXI). *R*_2_-weighted ^15^N-HSQC-AP was recorded with 256 scans each fid using an overall recycle delay of 200 ms, the in-phase experiment with 16 scans each fid and 4 s as recycle delay. Folded peaks are marked with an asterisk. **b** Expanded plot of selected rows of *R*_2_-weighted ^15^ N-HSQC-AP, corresponding to His7 and Asn27

As expected, the most representative spectra of the two *R*_2_ series are rather different. In Fig. 3a, the first time point of the exponential decay of the in-phase experiment is superimposed to the spectrum of the R_2_-weighted HSQC-AP series, recorded with *T* = 2.4 ms. Several signals, such as those of residues 22, 27–28, 36–37, 47–51, are indeed observable only in the *R*_2_-weighted HSQC-AP, while other residues are only barely visible in the first point of the exponential decay (e.g. residues 25 and 35). Fast relaxing signals are much better observed in the *R*_2_ weighted experiment, although signals are much broader than in the in-phase experiment, because they have been acquired as HN antiphase doublets. Like homonuclear ^1^H and ^13^C cases, a dispersion phase mode of the antiphase doublets produces the sum of the two dispersive components of opposite phase, thus giving rise to a pseudo-singlet with the maximum of signal intensity (Turner 1993; Bertini et al. 1994, 2005a). As an example, Fig. 3b shows selected rows for His7 and Asn27. In the case of His7, which has a negligible *R*_2para_ contribution, we observe that, when the signal linewidth is smaller than the splitting of the HN doublet, the typical pattern of an antiphase doublet phased in dispersion mode is observed; when signals are broader than the scalar coupling constant, like the case of Asn27 (*R*_2_ = 221 s^−1^), the doublet splitting is lost and the dispersive component of the doublet give rise to a well pseudo observable pseudo singlet. The removal of the inverse INEPT prevents transverse relaxation to be operative before ^1^H detection. Indeed, an additional refocusing would prevent the observation of signals characterized by *T*_2_ shorter than the INEPT period.

**Fig. 4:**
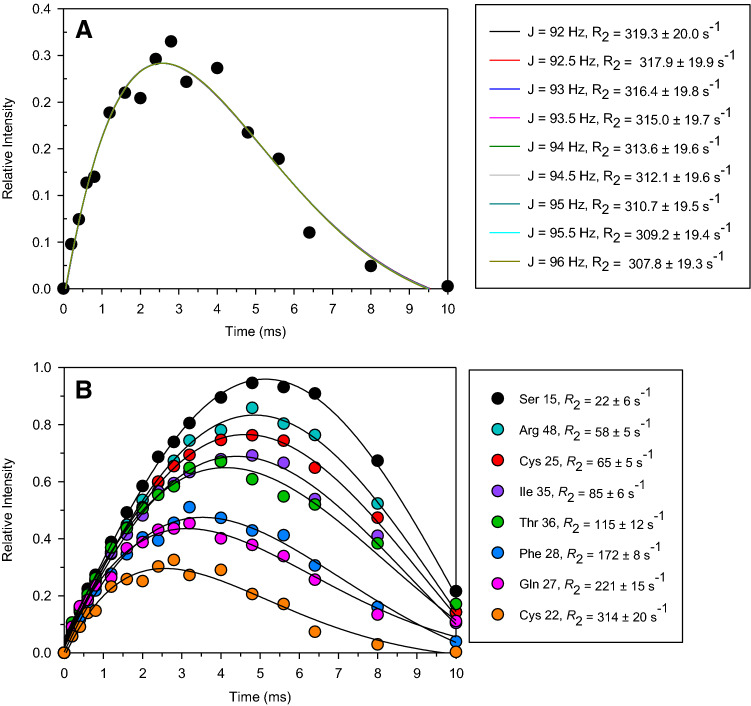
Peak intensities in the *R*_2_-weighted ^15^N-HSQC-AP experiment. Relative intensity are expressed with respect to I_0_ term of Eq. (2). **a** Buildup curves obtained with a two-parameter fitting of Eq. (2) using fixed *J* values. Fitted intensities are those of the R_2_-weighted ^15^N-HSQC-AP experiment for the case of Cys22 HN signal. When *J* was varied from 92 to 96 Hz, the interplay between *J* and *R*_2_ provide essentially the same best fitting curve, with *R*_2_ values in the range 308–319 s^−1^. The uncertainty of *R*_2_ due to the admissible values of *J* is smaller than the standard error (± 19.6 s^−1^) of each individual fitting. **b** Experimental build-up curves of some selected signals. Curve fitting using a two-parameter fit and *J* = 94 Hz give *R*_2_ values as indicated in Figure

Fitting of R_2_ values. The intensity of observed signals in the *R*_2_-weighted HSQC-AP can be analyzed according to:

2 $$ {\text{I}}\left( {\text{t}} \right) = {\mathbf{I}}_{{\mathbf{0}}} {\sin}({\text{p}}TJ){\exp}\left( { - T{\mathbf{R}}_{{\mathbf{2}}} } \right) $$

where *T* is the variable delay. In principle, a three-parameter fitting will provide values for **I**_**0**_, ***J*** and ***R***_***2***_, respectively representing the initial signal intensity, the ^1^J_HN_ scalar coupling constant, and the ^1^H *R*_2_ relaxation rate. However, the interplay between the two parameters ***J*** and ***R***_***2***_ is such that a three parameters fitting of the buildup curves tends either to over-estimate ***J*** and then compensate it with an under-evaluation of ***R***_***2***_ values or the other way around (smaller ***J*** and higher ***R***_***2***_ values). We therefore used a two-parameter fitting for Eq. (2), with constant *J* values. As shown in Fig. 4a, for *J* values in the range 92–96 Hz, i.e. the range of admissible values for ^1^J_HN_ scalar coupling at 500 MHz (residual dipolar couplings are expected to be negligible at 500 MHz for a protein of this size containing a [Fe_4_S_4_]^2+^ cluster), the deviations among calculated ***R***_***2***_ values were much smaller than the errors observed in each two-parameters fitting. We therefore decided to consider values and uncertainties taken from the two-parameter fitting obtained using *J* = 94 Hz as a fixed value. Some representative build-up curves are reported in Fig. 4b. The *R*_2_-weighted, ^15^ N-HSQC-AP experiment is able to measure *R*_2_ values for all HN signals of the protein, including those belonging to residues of the iron-bound cysteines, to residues H-bonded to cluster sulfide ions, and those spatially close to the 4Fe-4S cluster although not in direct electronic contact with the prosthetic group.

#### Data evaluation and assessment

**Fig. 5:**
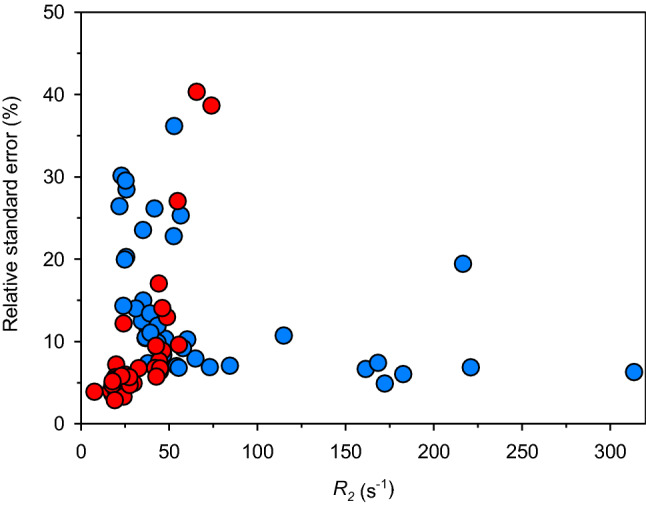
Transverse relaxation rates percentage errors (Δ*R*_2_/*R*_2_) obtained with the two series of experiments discussed in Fig. 2: (red) in-phase detected HSQC with relaxation delay preceding INEPT; (blue) *R*_2_-weighted HSQC-AP. The recycle delays for the two series were 4 s and 200 ms, respectively. For *R*_2_ values under 40 s^−1^, data from the in-phase experiment have much smaller errors than those from the *R*_2_-weighted HSQC-AP experiment. For *R*_2_ values over 50 s^−1^, the R_2_-weighted-HSQC-AP has much better performances. For *R*_2_ values above 80 s^−1^, only the R_2_-weighted-HSQC-AP provided reliable *R*_2_ measurements

The *R*_2_ values obtained with the *R*_2_-weighted HSQC-AP experiments are summarized in Table 1, together with those measured using the standard sequence. A data assessment is reported in Fig. 5, where, for both series, the value of the relative error (Δ*R*_2_/*R*_2_) vs *R*_2_ is shown. When *R*_2_ values are lower than 45 s^−1^, the accuracy of the sequence based on exponential decay and in-phase acquisition is much higher than that of the *R*_2_-weighted-HSQC-AP. Indeed, when INEPT (2τ_a_) and *R*_2_ relaxation (*T*) evolve into separate building blocks, a single exponential decay is measured and several methods allow one to measure *R*_2_ values with very good precision and accuracy (Iwahara et al. 2007). However, in the range 45–80 s^−1^ the relative errors become larger than those obtained with the *R*_2_-weighted-AP and, above 80 s^−1^, *R*_2_ values are measurable only (with the experimental conditions we used to perform the experiments) with the *R*_2_-weighted-AP sequence, up to *R*_2_ rates as large as 310 s^−1^. This indicates that, for signals with *R*_2_ rates larger than ca. 50 s^−1^, the *R*_2_-weighted-AP sequence should be the preferred method to measure relaxation rates, while the standard approach should be used in all other cases. Because all backbone HN signals of PioC have been identified and assigned and this was the fastest rate among them, it is not possible to set the upper limit threshold of the *R*_2_-weighted-HSQC-AP experiment. In principle (see Fig. 2b), one should be able to measure *R*_2_ values up to 400–500 s^−1^, provided that very broad signals do not fall within a crowded spectral region.

**Table 1:** 500 MHz, 298 K, observed transverse relaxation rates for PioC HN amide protons

δ^1^H (ppm)	δ^15^ N (ppm)	residue		R_2_ (s^−1^) AP	error (s^−1^) AP	R_2_ (s^−1^) IP	error (s^−1^) AP
		VAL	1				
8.26	117.2	THR	2			11.1	0.4
8.42	123.7	LYS	3			15.4	1.3
8.43	121.8	LYS	4	23.0	6.9	19.1	1.5
8.76	122.3	ALA	5	25.8	7.3	23.4	0.8
9.08	115.2	SER	6	38.8	5.2	17.8	0.6
9.64	116.3	HIS	7	37.8	4.4	21.3	0.8
8.49	116.5	LYS	8	52.9	19.1	32.8	2.2
8.13	119.5	ASP	9	35.3	5.3	16.9	0.7
8.23	118.2	ALA	10	34.6	4.3	19.6	0.8
7.69	105.0	GLY	11	46.8	3.9	20.0	1.4
8.19	116.3	TYR	12	36.6	3.8	29.9	1.5
8.67	124.5	GLN	13	54.3	3.8	46.4	4.1
8.22	115.2	GLU	14			19.4	1.1
7.29	112.8	SER	15	21.9	5.8	21.6	1.0
		PRO	16				
7.92	116.4	ASN	17	31.2	4.4	22.8	1.1
8.17	111.6	GLY	18	43.5	5.2	25.1	1.5
8.78	126.9	ALA	19	52.6	12.0	44.4	2.8
7.62	118.4	LYS	20	41.8	10.9	17.8	0.8
7.47	115.9	ARG	21	38.0	2.8	44.4	3.1
9.47	131.4	CYS	22	313.6	19.6	n.o	
8.62	97.5	GLY	23	60.3	6.2	48.8	6.3
8.62	109.8	THR	24	45.9	4.1	42.0	2.8
8.36	130.0	CYS	25	65.0	5.1	44.1	7.5
9.28	131.4	ARG	26	47.9	4.9	55.5	5.3
9.62	141.7	GLN	27	221.0	15.1	n.o	
7.72	123.4	PHE	28	172.3	8.4	n.o	
8.19	128.2	ARG	29	39.2	5.2	44.9	3.0
		PRO	30				
		PRO	31				
8.15	111.8	SER	32	43.5	5.2	25.1	1.5
7.55	114.9	SER	33	43.4	4.3	24.2	0.8
8.55	129.0	CYS	34	37.5	3.9	42.5	3.7
8.36	126.0	ILE	35	84.5	5.9	65.6	26
8.75	127.6	THR	36	115.0	12.3	n.o	
7.00	149.9	VAL	37	161.5	10.7	n.o	
8.61	122.8	GLU	38	43.0	4.0	24.2	0.8
8.43	115.7	SER	39	35.2	8.3	19.7	1.0
		PRO	40				
7.28	118.2	ILE	41	25.4	7.5	27.7	1.3
7.84	116.4	SER	42	36.7	3.8	42.8	2.4
9.12	121.2	GLU	43	24.1	3.4	19.2	0.5
7.98	116.8	ASN	44	25.7	5.2	27.4	1.5
7.20	102.6	GLY	45	55.5	3.8	46.2	6.5
7.86	113.7	TRP	46	73.1	5.0	54.8	14.8
6.47	118.9	CYS	47	182.7	11.0	n.o	
6.79	115.2	ARG	48	57.9	5.3	74.0	28.6
7.74	154.7	LEU	49	168.4	12.4	n.o	
6.02	119.5	TYR	50	56.6	14.3	n.o	
5.46	119.0	ALA	51	216.6	42.0	n.o	
8.33	109.7	GLY	52	39.4	4.4	23.0	1.3
8.43	120.2	LYS	53	24.9	5.0	18.0	0.9
8.04	128.5	ALA	54			7.7	0.3
8.53	124,0	TRP sc	46	53.1	4.2	33.9	2.1

Columns 5–6 refer to values observed with the *R*_2_-weighted HSQC-AP experiment, columns 7–8 to values observed with an in-phase HSQC experiment with a single exponential *T*_2_ decay prior to INEPT

The poor agreement between the two sets of values obtained with the two experiments and reported in Table 1 also deserves a comment. Indeed, the *R*_2_-weighted HSQC-AP experiment has been optimized to measure *R*_2_ rates of fast relaxing signals, which are the target of this experiment. To this end, we have used, for the *R*_2_-weighted HSQC-AP, recycle delays that are a factor 20 shorter than those used in the in-phase sequence (200 ms vs 4 s). Due to the fast repetition of the experiment, signals with *R*_2_ < 35 s^−1^ suffer from partial saturation. As a matter of speculation, it would always be possible to perform the *R*_2_-weighted HSQC-AP experiment with longer recycle delays to properly fit *R*_2_ values of slower relaxing signals. However, the obvious complementarity between the two experiments, given by the time scale of the recoveries measurable with the two experiments, and the intrinsic higher precision of the in phase experiment provided by the single exponential dependence, suggest that a combination of two *R*_2_ measurements, respectively optimized for the quantification of small and large PREs would be, by far, the most efficient approach.

## Conclusions

In summary, the R_2_-weighted HSQC-AP experiment, proposed here, is the simplest experiment for *R*_2_ relaxation, in which ^1^H magnetization is kept along the transverse plane only during the relaxation delay and *t*_2_ acquisition and all periods of *J*_HN_ evolution/refocusing are removed. This simplified scheme not only detects signals that escape detection in a conventional HSQC experiment, but measures *R*_2_ values that are fully reliable, in the range of rates 50–400 s^−1^. This is extremely important for metalloproteins, where the first coordination sphere of the metal ion(s) and its immediate neighboring regions can be identified, characterized and, finally, restrained into NMR structure calculations only when it is possible to obtain paramagnetism-based structural restraints such as PREs. The small HiPIP protein PioC is a challenging and significant example of the application of this experiment, where this objective was achieved and the *R*_2_ could be measured for all four cysteines coordinating the paramagnetic cluster. Finally, it is worth noting that this approach can be useful in all other cases where R_2_ is larger than 50 s^−1^: conformation dynamics and exchange phenomena often increase relaxation rates above this threshold also in diamagnetic systems, thus extending the potential applications of this approach.

## References

[CR100] Ab E, Atkinson AR, Banci L, Bertini I, Ciofi-Baffoni S, Brunner K, Diercks T, Doetsch V, Engelke F, Folkers G, Griesinger C, Gronwald W, Gunther H, Habeck M, de Jong R, Kalbitzer HR, Kieffer B, Leeflang BR, Loss S, Luchinat C, Marquardsen T, Moskau D, Neidig KP, Nilges M, Piccioli M, Pierattelli R, Rieping W, Schippmann T, Schwalbe H, Trave G, Trenner JM, Wohnert J, Zweckstetter M, Kaptein R (2006). NMR in Structural Proteomics. Acta Crystallogr D Biol Crystallogr.

[CR1] Anthis NJ, Clore GM (2015). Visualizing transient dark states by NMR spectroscopy. Q Rev Biophys.

[CR2] Arnesano F (2003). A strategy for the NMR characterization of type II copper(II) proteins: the case of the copper trafficking protein CopC from *Pseudomonas syringae*. J Am Chem Soc.

[CR3] Arnesano F, Banci L, Piccioli M (2006). NMR structures of paramagnetic metalloproteins. Q Rev Biophys.

[CR4] Balayssac S, Jiménez B, Piccioli M (2006). Assignment strategy for fast relaxing signals: complete aminoacid identification in thulium substituted calbindin D_9k_. J Biomol NMR.

[CR5] Banci L, Bertini I, Luchinat C (1990). The ^1^H NMR parameters of magnetically coupled dimers - the Fe_2_S_2_ proteins as an example. Struct Bonding.

[CR6] Banci L, Camponeschi F, Ciofi-Baffoni S, Piccioli M (2018). The NMR contribution to protein-protein networking in Fe-S protein maturation. J Biol Inorg Chem.

[CR7] Battiste JL, Wagner G (2000). Utilization of site-directed spin labelling and high-resolution heteronuclear nuclear magnetic resonance for global fold determination of large proteins with limited Nuclear Overhauser Effect data. Biochemistry.

[CR8] Bermel W (2005). Complete assignment of heteronuclear protein resonances by protonless NMR spectroscopy. Angew Chem Int Ed.

[CR9] Bertini I (1992). Identification of the iron ions of HiPIP from *Chromatium vinosum* within the protein frame through 2D NMR experiments. J Am Chem Soc.

[CR10] Bertini I, Capozzi F, Luchinat C, Piccioli M, VicensOliver M (1992). NMR is a unique and necessary step in the investigation of iron-sulfur proteins: the HiPIP from *R. gelatinosus* as an example. Inorg Chim Acta.

[CR11] Bertini I, Donaire A, Luchinat C, Rosato A (1997). Paramagnetic relaxation as a tool for solution structure determination: *Clostridium pasterianum* ferredoxin as an example. Proteins Struct Funct Genet.

[CR12] Bertini I, Jiménez B, Piccioli M (2005). ^13^C direct detected experiments: optimisation to paramagnetic signals. J Magn Reson.

[CR103] Bertini I, Jiménez B, Piccioli M, Poggi L (2005). Asymmetry in C−C COSY spectra provides information on ligand geometry in paramagnetic proteins. J Am Chem Soc.

[CR13] Bertini I, Luchinat C, Parigi G, Ravera E (2016). NMR of paramagnetic molecules.

[CR14] Bertini I, Luchinat C, Piccioli M, Tarchi D (1994). COSY spectra of paramagnetic macromolecules, observability, scalar effects, cross correlation effects, relaxation allowed coherence transfer. Concepts Magn Reson.

[CR15] Bertini I, Luhat C (1986). NMR of paramagnetic molecules in biological systems.

[CR16] Bird LJ (2014). Nonredundant roles for cytochrome c2 and two high-potential iron-sulfur proteins in the photoferrotroph Rhodopseudomonas palustris TIE-1. J Bacteriol.

[CR17] Blondin G, Girerd J-J (1990). Interplay of electron exchange and electron transfer in metal polynuclear complexes in proteins or chemical models. Chem Rev.

[CR18] Brancaccio D (2017). [4Fe-4S] Cluster Assembly in Mitochondria and Its Impairment by Copper. J Am Chem Soc.

[CR19] Camponeschi F, Muzzioli R, Ciofi-Baffoni S, Piccioli M, Banci L (2019). Paramagnetic (1)H NMR Spectroscopy to Investigate the Catalytic Mechanism of Radical S-Adenosylmethionine Enzymes. J Mol Biol.

[CR20] Cetiner EC (2019). Paramagnetic-iterative relaxation matrix approach: extracting PRE-restraints from NOESY spectra for 3D structure elucidation of biomolecules. J Biomol NMR.

[CR21] Cheng H, Markley JL (1995). NMR spectroscopic studies of paramagnetic proteins: iron-sulfur proteins. Annu Rev Biophys Biomol Struct.

[CR22] Ciofi-Baffoni S, Gallo A, Muzzioli R, Piccioli M (2014). The IR-N-15-HSQC-AP experiment: a new tool for NMR spectroscopy of paramagnetic molecules. J Biomol NMR.

[CR23] Clore GM (2015). Practical aspects of paramagnetic relaxation enhancement in biological macromolecules. Methods Enzymol.

[CR24] Clore GM, Iwahara J (2009). Theory, practice, and applications of paramagnetic relaxation enhancement for the characterization of transient low-population states of biological macromolecules and their complexes. Chem Rev.

[CR25] Donaldson LW (2001). Structural characterization of proteins with an attached ATCUN Motif by paramagnetic relaxation enhancement NMR spectroscopy. J Am Chem Soc.

[CR26] Hansen DF, Led JJ (2006). Determination of the geometric structure of the metal site in a blue copper protein by paramagnetic NMR. Proc Natl Acad Sci USA.

[CR27] Harford C, Sarkar B (1997). Amino Terminal Cu(II)- and Ni(II)-Binding (ATCUN) Motif of Proteins and Peptides: Metal Binding, DNA Cleavage, and Other Properties. Acc Chem Res.

[CR28] Hass MAS, Ubbink M (2014). Structure determination of protein-protein complexes with long-range anisotropic paramagnetic NMR restraints. Curr Opin Struct Biol.

[CR29] Helms G, Satterlee JD (2013). Keeping PASE with WEFT: SHWEFT-PASE pulse sequences for H-1 NMR spectra of highly paramagnetic molecules. Magn Reson Chem.

[CR30] Inubushi T, Becker ED (1983). Efficient detection of paramagnetically shifted NMR resonances by optimizing the WEFT pulse sequence. J Magn Reson.

[CR31] Iwahara J, Clore GM (2010). Structure-independent analysis of the breadth of the positional distribution of disordered groups in macromolecules from order parameters for long, variable-length vectors using NMR paramagnetic relaxation enhancement. J Am Chem Soc.

[CR32] Iwahara J, Schwieters CD, Clore GM (2004). Ensemble approach for NMR structure refinement against H-1 paramagnetic relaxation enhancement data arising from a flexible paramagnetic group attached to a macromolecule. J Am Chem Soc.

[CR33] Iwahara J, Tang C, Clore GM (2007). Practical aspects of ^1^H transverse paramagnetic relaxation enhancement measurements on macromolecules. J Magn Reson.

[CR34] Jensen MR, Lauritzen C, Dahl SW, Pedersen J, Led JJ (2004). Binding ability of a HHP-tagged protein towards Ni^2+^ studied by paramagnetic NMR relaxation: the possibility of obtaining long-range structure information. J Biomol NMR.

[CR35] Joss D, Haussinger D (2019). Design and applications of lanthanide chelating tags for pseudocontact shift NMR spectroscopy with biomacromolecules. Prog Nucl Magn Reson Spectrosc.

[CR36] Kateb F, Piccioli M (2003). New routes to the detection of relaxation allowed coherence transfer in paramagnetic molecules. J Am Chem Soc.

[CR37] Keizers PHJ, Ubbink M (2011). Paramagnetic tagging for protein structure and dynamics analysis. Prog Nucl Magn Reson Spectrosc.

[CR38] Koehler J, Meiler J (2011). Expanding the utility of NMR restraints with paramagnetic compounds: background and practical aspects. Prog Nucl Magn Reson Spectrosc.

[CR39] Kolczak U, Salgado J, Siegal G, Saraste M, Canters GW (1999). Paramagnetic NMR studies of blue and purple copper proteins. Biospectroscopy.

[CR40] Kudhair BK (2017). Structure of a Wbl protein and implications for NO sensing by *M. tuberculosis*. Nat Commun.

[CR41] Lin IJ (2009). Hyperfine-shifted ^13^C and ^15^N NMR signals from *Clostridium pasteurianum* Rubredoxin: extensive assignments and quantum chemical verification. J Am Chem Soc.

[CR42] Lin I, Gebel EB, Machonkin TE, Westler WM, Markley JL (2003). Correlation bewtween hydrogen bond lenghts and reduction potentials in Clostridium Pasteurianum Rubredoxin. J Am Chem Soc.

[CR43] Liu W-M, Overhand M, Ubbink M (2014). The application of paramagnetic lanthanoid ions in NMR spectroscopy on proteins. Coord Chem Rev.

[CR44] Machonkin TE, Westler WM, Markley JL (2002). ^13^C–^13^C 2D NMR: a novel strategy for the study of paramagnetic proteins with slow electronic relaxation times. J Am Chem Soc.

[CR45] Machonkin TE, Westler WM, Markley JL (2005). Paramagnetic NMR spectroscopy and density functional calculations in the analysis of the geometric and electronic structures of iron-sulfur proteins. Inorg Chem.

[CR46] Madl T, Felli IC, Bertini I, Sattler M (2010). Structural analysis of protein interfaces from 13C direct-detect paramagnetic relaxation enhancements. J Am Chem Soc.

[CR47] Matei E, Gronenborn AM (2016). (19)F paramagnetic relaxation enhancement: a valuable tool for distance measurements in proteins. Angew Chem Int Ed Engl.

[CR48] Mateos B, Konrat R, Pierattelli R, Felli IC (2019). NMR characterization of long-range contacts in intrinsically disordered proteins from paramagnetic relaxation enhancement in C-13 direct-detection experiments. ChemBioChem.

[CR49] Miao Q (2019). A double-armed, hydrophilic transition metal complex as a paramagnetic NMR Probe. Angew Chem Int Ed Engl.

[CR101] Mori M, Jiménez B, Piccioli M, Battistoni A, Sette M (2008). The solution structure of the monomeric copper, zinc superoxide dismutase from *Salmonella enterica*: structural insights to understand the evolution toward the dimeric structure. Biochemistry.

[CR50] Mori M, Kateb F, Bodenhausen G, Piccioli M, Abergel D (2010). Towards structural dynamics: protein motions viewed by chemical shift modulations and direct detection of C'N multiple-quantum relaxation. J Am Chem Soc.

[CR51] Mouesca J-M, Lamotte B (1998). Iron-Sulfur clusters and their electronic and magnetic properties. Coord Chem Rev.

[CR52] Nitsche C, Otting G (2017). Pseudocontact shifts in biomolecular NMR using paramagnetic metal tags. Prog Nucl Magn Reson Spectrosc.

[CR53] Parigi G, Ravera E, Luchinat C (2019). Magnetic susceptibility and paramagnetism-based NMR. Prog Nucl Magn Reson Spectrosc.

[CR54] Phillips WD, Poe M, McDonald CC, Bartsch RG (1970). Proc Natl Acad Sci USA.

[CR55] Piccioli M, Turano P (2015). Transient iron coordination sites in proteins: exploiting the dual nature of paramagnetic NMR. Coord Chem Rev.

[CR57] Pintacuda G (2004). Fast structure /based assignment of ^15^N HSQC spectra of selectively ^15^N labeled paramagnetic proteins. J Am Chem Soc.

[CR58] Pintacuda G, Hohenthanner K, Otting G, Muller N (2003). Angular dependence of dipole-dipole-Curie-spin cross-correlation effects in high-spin and low-spin paramagnetic myoglobin. J Biomol NMR.

[CR59] Pintacuda G, John M, Su XC, Otting G (2007). NMR structure determination of protein-ligand complexes by lanthanide labeling. Acc Chem Res.

[CR60] Sato K, Kohzuma T, Dennison C (2003). Active-site structure and electron-transfer reactivity of plastocyanins. J Am Chem Soc.

[CR61] Siegal G, Selenko P (2019). Cells, drugs and NMR. J Magn Reson.

[CR62] Softley CA, Bostock MJ, Popowicz GM, Sattler M (2020). Paramagnetic NMR in drug discovery. J Biomol NMR.

[CR63] Solomon I (1955). Relaxation processes in a system of two spins. Phys Rev.

[CR64] Spronk C (2018). Structure and dynamics of Helicobacter pylori nickel-chaperone HypA: an integrated approach using NMR spectroscopy, functional assays and computational tools. J Biol Inorg Chem.

[CR65] Tang C, Iwahara J, Clore GM (2006). Visualization of transient encounter complexes in protein-protein association. Nature.

[CR56] Trindade IB, Invernici M, Cantini F, Louro RO, Piccioli M (2020). ^1^H, ^13^C and ^15^N assignment of the paramagnetic high potential iron-sulfur protein (HiPIP) PioC from *Rhodopseudomonas palustris* TIE-1. Biomol NMR Assign.

[CR66] Trindade I, Invernici M, Cantini F, Louro RO, Piccioli M (2020b) NOE-less protein NMR structures-an alternative approach in Highly Paramagnetic Systems. arXiv: 2002.01228 10.1111/febs.15615 33124176

[CR67] Turner DL (1993). Optimization of COSY and related methods. Applications to 1H NMR of horse ferricytochrome c. J Magn Reson Ser A.

[CR68] Turner DL, Brennan L, Chamberlin SG, Louro RO, Xavier AV (1998). Determination of solution structures of paramagnetic proteins by NMR. Eur Biophys J.

